# A Rare Case of Lithium-induced Partial Nephrogenic Diabetes Insipidus

**DOI:** 10.7759/cureus.7877

**Published:** 2020-04-28

**Authors:** Abdul Rauf, Sawara Gul, Mohammad Nasir, Uroosa Arif, Mosunmoluwa Oyenuga

**Affiliations:** 1 Internal Medicine, SSM Health St. Mary's Hospital, St. Louis, USA; 2 Internal Medicine, Lady Reading Hospital, Peshawar, PAK; 3 Neurological Surgery, Hayatabad Medical Complex, Peshawar, PAK; 4 Internal Medicine, Khyber Teaching Hospital, Peshawar, PAK

**Keywords:** lithium induced diabetes insipidus, diabetes insipidus, partial diabetes insipidus, lithium induced partial diabetes insipidus

## Abstract

Chronic lithium therapy in patients with bipolar disorder and other psychiatric illnesses can lead to a very common side effect of complete or partial nephrogenic diabetes insipidus. After confirmation of hypotonic polyuria, water deprivation test with desmopressin injection is used to make the diagnosis. Incomplete response to desmopressin suggests partial nephrogenic diabetes insipidus. We report a rare case of a 58-year-old patient who presented with hypernatremia and hypotonic polyuria secondary to chronic lithium therapy. She was diagnosed with partial nephrogenic diabetes insipidus secondary to chronic lithium therapy and was treated with amiloride resulting in improvement.

## Introduction

Lithium is widely used to treat patients with mood disorders like bipolar disorder. Chronic lithium therapy can lead to accumulation in distal tubular cells causing impaired urinary concentrating ability. This can lead to partial or full nephrogenic diabetes insipidus. It is estimated to be present in up to 40% of patients on chronic lithium therapy [1]. Diabetes insipidus is a syndrome characterized by the excretion of large volumes of dilute urine. The diagnosis is confirmed with water deprivation test followed by desmopressin injection. Partial diabetes insipidus is characterized by an incomplete response to desmopressin. We report a case of lithium-induced partial nephrogenic diabetes insipidus.

## Case presentation

**Figure 1:**
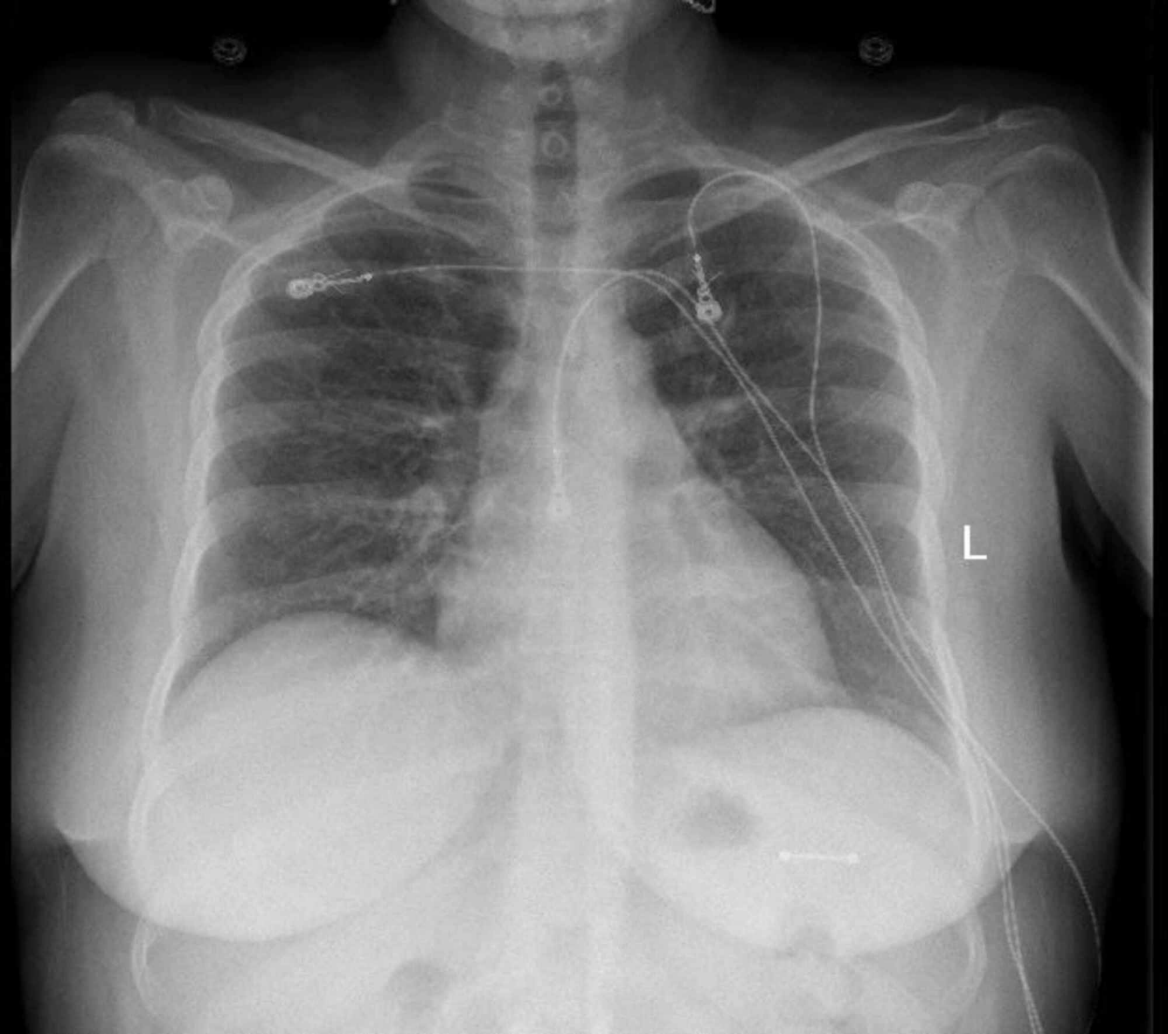
X-ray of the chest

**Figure 2:**
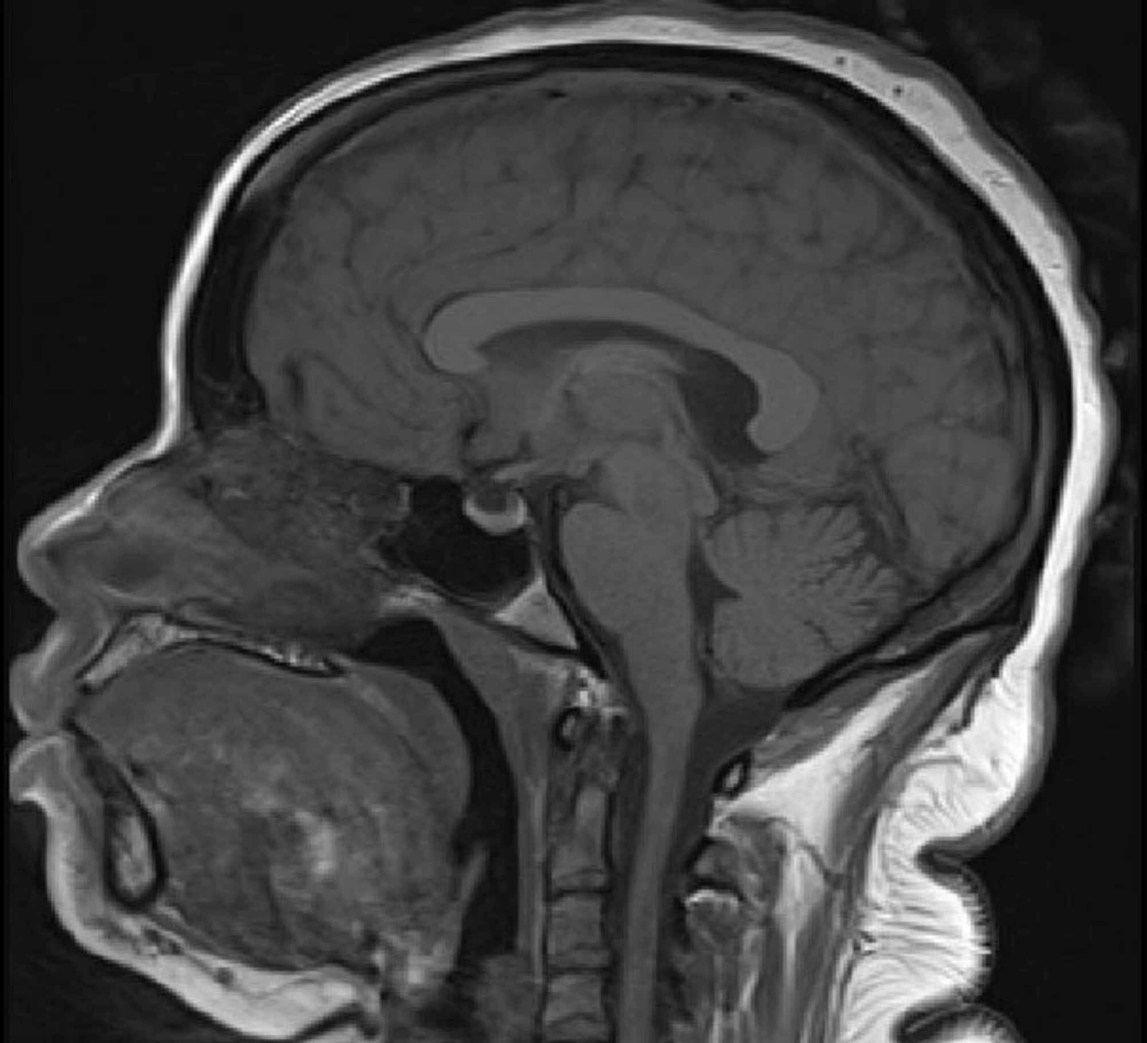
T1-weighted sagittal MRI of the brain

A 58-year-old female with a past medical history of bipolar disorder presented to the emergency department with acute onset of dyspnea, upper respiratory symptoms, restlessness, and agitation. According to the family, the patient had been on lithium therapy for the past nine years. There was no family history of any endocrine or renal diseases. On examination, her blood pressure was 156/76 mmHg, respiratory rate of 23/min, saturation 91% on room air, and a pulse rate of 101/min. The chest was clear on auscultation with no added sounds. Blood work showed a hemoglobin of 12.1 g/dL (normal: 14 - 17 g/dL), hematocrit - 45.2% (normal: 41% - 51%), WBC - 13 K/uL (80% neutrophils) (normal: 3.3 - 8.7 K/uL), platelets - 170 K/uL (normal: 147 - 347 K/uL), calcium - 9.4 mg/dL (normal: 9 - 10.5 mg/dL), blood urea nitrogen (BUN) - 26 mg/dL (normal: 8 - 20 mg/dL), creatinine - 1.1 mg/dL (normal: 0.7 - 1.3 mg/dL), sodium - 151 mmol/L (normal: (136 - 145 mmol/L), and lithium - 0.9 (therapeutic range (0.8 - 1.2). Respiratory pathogen viral polymerase chain reaction (PCR) was positive for influenza. The chest X-ray was normal (Figure 1). MRI brain did not reveal any underlying pathology (Figure 2). Due to increasing agitation, she was intubated for airway protection and started on Tamiflu for influenza. She was transferred to the intensive care unit for further management. On day two of follow-up, the patient was found to have hypotonic polyuria with a 24-urine output of 7L, urine osmolality of 316 mOsm/kg, with serum sodium of 151 mEq/L and serum osmolality of 327 mOsm/kg (Table 1). As her serum osmolality (327 mOsm/kg), and serum sodium (151 mEq/L) were above the threshold for maximal arginine vasopressin (AVP) secretion, a water deprivation test was not performed. Thus, we performed a desmopressin challenge test on day two. After desmopressin injection, her urine osmolality increased to 485 mOsm/kg, which was approximately 35% increase from baseline, less than 50% increase indicating partial nephrogenic diabetes insipidus (Table 2). As lithium could not be discontinued in the patient (she had difficulty controlling bipolar disorder per psychiatry), she was started on amiloride for lithium-induced partial nephrogenic diabetes insipidus. On day three of follow-up, she was extubated. Subsequently, her hypernatremia resolved in the next three days with amiloride therapy. Serum sodium normalized to 141 mmol/L and urine output decreased to 2340 ml/24 hours. She was subsequently discharged to a long term care facility.

**Table 1:** Serum chemistry and urine volume in the patient with lithium-induced partial diabetes insipidus, on amiloride therapy

Day of admission	Serum Sodium (mmol/L)	Urine volume (ml/24 hours)	Serum osmolality (mOsm/Kg)
Day 1	151	7000	327
Day 2	148	6100	315
Day 3	148	5350	312
Day 4	144	3620	298
Day 5	141	2340	292

**Table 2:** Urine osmolality before and after desmopressin administration

Before desmopressin administration	316 mOsm/kg
After desmopressin administration	485 mOsm/Kg

## Discussion

Lithium is one of the mainstays of treatment for bipolar spectrum disorders. The most frequent side effect of lithium is nephrogenic diabetes insipidus, which occurs in up to 40% of patients [1].

Diabetes insipidus is characterized by the excretion of large amounts of hypotonic urine. It is caused by either a deficiency of hormone AVP in the pituitary/hypothalamus called central diabetes insipidus or resistance to the actions of AVP in the kidneys called nephrogenic diabetes insipidus. Sometimes the accurate diagnosis of diabetes insipidus can be challenging as there is overlap between different forms of diabetes insipidus in terms of results obtained from diagnostic testing. For diagnoses of diabetes insipidus, the initial step is to confirm the presence of hypotonic polyuria. Polyuria is defined as excretion of urinary volume >50 ml/Kg/24 hours in adults [2]. Then the water deprivation test is performed to distinguish diabetes insipidus from primary polydipsia. Once diabetes insipidus is confirmed, it is further classified into central diabetes insipidus and nephrogenic diabetes insipidus by desmopressin administration. An increase of at least >50% in urine osmolality after desmopressin administration points towards central diabetes insipidus, while <50% increase in urine osmolality after desmopressin administration suggests nephrogenic diabetes insipidus [3]. In some patients, partial diabetes insipidus is diagnosed, when there is an increase in urine osmolality over 300 mOsm/Kg prior to an increase in serum sodium/plasma osmolality. In such cases, it means some endogenous vasopressin activity or renal response to vasopressin is present. With partial nephrogenic diabetes insipidus, the urine osmolality after water deprivation is usually between 300-800 mOsm/kg and there is a <50% increase in urine osmolality after desmopressin administration [4].

Patients who are on chronic lithium therapy can develop resistance to antidiuretic hormone (ADH) that results in nephrogenic diabetes insipidus. ADH acts on the principal cells in the collecting tubules regulating the water permeability. It causes the fusion of aquaporin-2 (AQP2) water channels with the luminal membrane in principal cells, causing water absorption. Lithium enters the principal cells through epithelial sodium channels in the luminal membrane and affects the ADH function on the cells [1]. Several mechanisms have been suggested by which lithium interferes with ADH function. These include reducing AQP2 water channels by degradation and reducing AQP2 gene transcription decreasing the number of principal cells [5-7].

Amiloride is considered one of the first-line medications to treat lithium-induced nephrogenic diabetes insipidus, particularly in those patients who need to be on lithium therapy despite the development of nephrogenic diabetes insipidus. Amiloride acts by blocking lithium entry into collecting tubules by closing sodium channels and so lithium cannot interfere in the function of ADH in these cells [8].

As was the case with our patient, her urine osmolality was 316 mOsm/Kg before the administration of desmopressin and the increase in osmolality was <50% afterward. A diagnosis of lithium-induced partial nephrogenic diabetes insipidus was made based on the results of baseline osmolality and electrolytes, poor response to desmopressin, and no identifiable etiology of central causes of diabetes insipidus, and in the context of the patient being on chronic lithium therapy for a duration of nine years. The patient also responded well to therapy with amiloride, the first line of treatment considered for lithium-induced nephrogenic diabetes insipidus [9]. On a thorough review of literature, only one case of partial nephrogenic diabetes insipidus secondary to chronic lithium therapy has been described so far [10]. Clinicians should be able to recognize cases of lithium-induced diabetes insipidus. It is important to identify partial nephrogenic diabetes insipidus in patients on lithium as it responds better to amiloride compared to full nephrogenic diabetes insipidus [10].

## Conclusions

Partial nephrogenic diabetes insipidus secondary to chronic lithium therapy is a rare phenomenon. Chronic lithium therapy is associated with an impaired urinary concentrating ability and may result in partial or complete nephrogenic diabetes insipidus. In difficult to treat psychiatric illness where lithium cessation is not ideal, partial nephrogenic diabetes insipidus can be treated with amiloride that can restore the urinary concentrating ability. Clinicians should be able to recognize cases of lithium-induced diabetes insipidus and identify partial nephrogenic diabetes insipidus in patients on lithium as it responds better to amiloride compared to full nephrogenic diabetes.
